# Biosurfactants: Promising Molecules for Petroleum Biotechnology Advances

**DOI:** 10.3389/fmicb.2016.01718

**Published:** 2016-10-31

**Authors:** Darne G. De Almeida, Rita de Cássia F. Soares Da Silva, Juliana M. Luna, Raquel D. Rufino, Valdemir A. Santos, Ibrahim M. Banat, Leonie A. Sarubbo

**Affiliations:** ^1^Northeast Biotechnology Network (RENORBIO), Federal Rural University of PernambucoRecife, Brazil; ^2^Advanced Institute of Technology and InnovationRecife, Brazil; ^3^Center of Sciences and Technology, Catholic University of Pernambuco (UNICAP)Recife, Brazil; ^4^Faculty of Life and Health Sciences, School of Biomedical Sciences, University of UlsterUlster, UK

**Keywords:** biosurfactants, petroleum biotechnology, emulsified fuels, enhanced oil recovery, bitumen, sulfate reducing bacteria

## Abstract

The growing global demand for sustainable technologies that improves the efficiency of petrochemical processes in the oil industry has driven advances in petroleum biotechnology in recent years. Petroleum industry uses substantial amounts of petrochemical-based synthetic surfactants in its activities as mobilizing agents to increase the availability or recovery of hydrocarbons as well as many other applications related to extraction, treatment, cleaning, and transportation. However, biosurfactants have several potential applications for use across the oil processing chain and in the formulations of petrochemical products such as emulsifying/demulsifying agents, anticorrosive, biocides for sulfate-reducing bacteria, fuel formulation, extraction of bitumen from tar sands, and many other innovative applications. Due to their versatility and proven efficiency, biosurfactants are often presented as valuable versatile tools that can transform and modernize petroleum biotechnology in an attempt to provide a true picture of state of the art and directions or use in the oil industry. We believe that biosurfactants are going to have a significant role in many future applications in the oil industries and in this review therefore, we highlight recent important relevant applications, patents disclosures and potential future applications for biosurfactants in petroleum and related industries.

## Introduction

Petroleum is the most important energy resource and raw material for the chemical industry and has driven the development of the modern world and global intensive economic development for the past century (Okoliegbe and Agarry, 2012; Silva et al., 2014). We depend on it for our basic needs for heat, light and transportation. Prediction of the world energy demand indicates a 1.7% annual increase in the number of oil barrels produced annually between the years 2000 to 2030, while oil consumption is expected to reach 15.3 billion tons annually. If current levels of world consumption are maintained the oil reserves available can allow meeting these demand for approximately 40 years (Elraies and Tan, 2012; Silva et al., 2014). There is no an energy source available at present that could meet or compete with oil, making the largest energy consumers dependent on countries with large oil reserves (Elraies and Tan, 2012). The US Department of Energy for example, reported that the majority (≈83.0%) of primary energy sources within the US are fossil fuels derived, of which 57.0% are from petroleum products. In 2010 19.2 million cubic meters of petroleum were consumed on daily basis (Santos et al., 2016).

The USA produces 870,000 m^3^ of crude oil on daily basis from 530,000 production-wells, the majority of which produce ≤1.59 m^3^, therefore high quality easily extractable light crude oils are limited and poses two major issues: first, efficiency and maximization of the overall stages of processing and secondly, the ability to utilize the heavy crude oils, bitumen and tar-sand components (Santos et al., 2016). On the whole petroleum production has been steadily moving toward the extraction of heavy/extra-heavy oils rather than medium to light oils, according to the International Energy Agency. In countries such as China, Canada, Venezuela, Mexico, and the USA; the heavy crude oils represent approximately half of recoverable oil resources. The development of efficient uses for this resource therefore is fast becoming an important technology (Cerón-Camacho et al., 2013).

Petroleum biotechnology has become an emerging technology that aims to implement biological processes to explore, produce, transform, and refine petroleum to generate valuable by-products and to reduce, manage and clean any pollution output and to treat petroleum industrial effluents (Silva et al., 2014). The versatility of microbes and microbial metabolism and their intrinsic ability to mediate transformation of complex raw materials at a wide range under extreme conditions such as high salinity, temperature, pH values, pressure, and hydrophobicity, facilitates the development of these technologies (Montiel et al., 2009). Among the emerging biotechnologies with application prospects in the oil industry, those using biosurfactants have stood out promisingly (Silva et al., 2014).

Biosurfactants are expected to become known as multifunctional materials of the twenty first century as they have applications in different industrial processes as well as potential novel future uses (Marchant and Banat, 2012) mostly due to their diverse structures. Microorganisms produce surface active compounds to enhance both the bioavailability of hydrophobic immiscible and mostly inaccessible substrates allowing better survival under low moisture conditions. Biosurfactant production generally requires the presence of miscible hydrophilic and oily/hydrocarbon type carbon source in the culture medium. The process economics and environmental credentials can make it attractive when using waste products as substrates (Makkar et al., 2011; Dziegielewska and Adamczak, 2013). Currently, the major emerging market for biosurfactants has been the petroleum related industries to allow effective exploration of heavy oil, offering advantages over chemical surfactants in processes involving extraction, transportation, storage and refining. Biosurfactants have also been successfully used in cleaning of oil sludge in storage tanks, microbial-enhanced oil recovery and to facilitate better transportation of heavy crude oil though pipeline (Assadi and Tabatabaee, 2010; Luna et al., 2012; Sobrinho et al., 2013).

This review discuss biosurfactants potential roles and applications within the petroleum industry. Roles in processes of petroleum exploration, treatment, transport, and remediation as well as patents disclosures related to biosurfactants application by petroleum industry and related market trends and future potentials are all described in details.

## Petroleum biotechnology

Petroleum is believed to have originated from the organic matter of microorganisms and algae that form the plankton deposited over millennia, which did not undergo oxidation process and accumulated in the bottom of the primitive oceans and was covered by sediment. The interaction between the organic matter, sediments and appropriate thermochemical conditions was fundamental to the beginning of the chain of processes which led to the formation of petroleum (Thomas, 2004). Crude oil usually consists of two or three different components/phases (namely gas, liquid and solid). The petroleum industry uses several separation mechanisms to separate these from one another (Holmager, 2010).

Exploration includes prospecting, seismic and drilling activities (Devold, 2013). Primary recovery mainly uses the reservoir's natural innate energy to displace oil from the porous rocks (Elraies and Tan, 2012) while conventional secondary recovery method involves water and/or gas injections to increase oil displacement, mobility and productivity of the oil well. A significant proportion of crude oil (>50%) however are often unrecoverable by conventional oil recovery methods and remains trapped in reservoirs (Bachmann et al., 2014). Ways to further increasing oil production are often carried out through tertiary enhanced oil recovery (EOR) methods which may result in recovering significant additional portions of the oil remaining after conventional methods (Elraies and Tan, 2012). Petroleum refining, on the other hand, are traditionally based on the use of physicochemical processes including chemical catalysis and distillation that operates under high pressures and temperatures where the crude oil and condensate are processed into a multitude of marketable products with defined specifications, such as gasoline, diesel fuel or raw material for the petrochemical industry (Singh et al., 2012; Devold, 2013).

**Figure 1:**
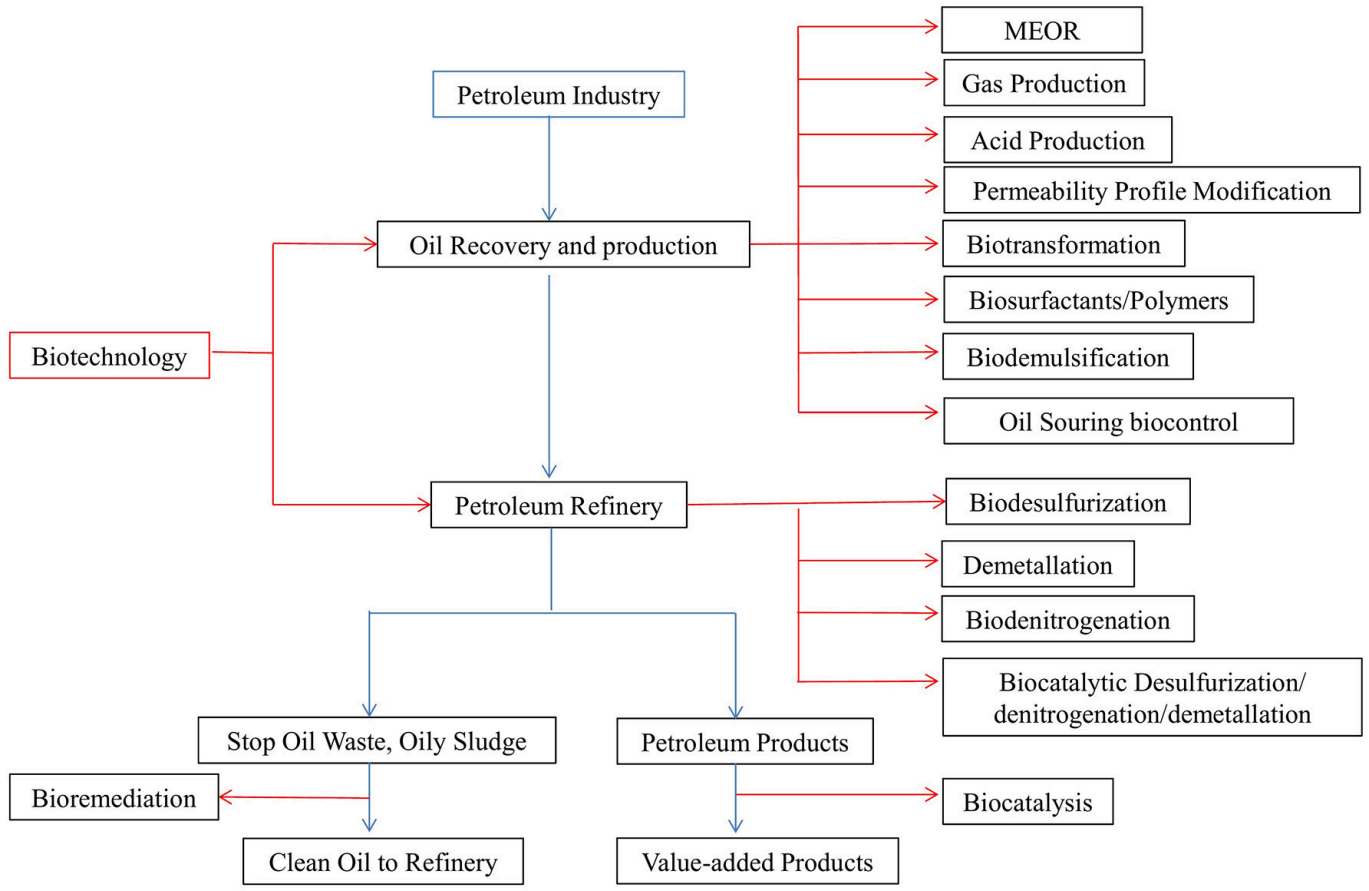
**Potential applications of biotechnology in petroleum industry**. Blue lines represent the major petroleum processing steps, and the red lines represent the biotechnological applications in the respective steps.

Biotechnology has played a significant role in enhancing crude oil recovery from depleted oil reservoirs and as a tool to increase stagnant petroleum production as well as in the refining and processing and further managing environmentally safe pollutant remediation and disposal practices (Sen, 2008; Singh et al., 2012). The use of bioprocess in this industry has expanded to the application of technologies related to biodesulfurization, biodemetallation, biodenitrogenation, and biotransformation and into crude oil refining associated with upgrading of fuels, production of fine chemicals, reduction of souring during production, complementing techniques such as microbial enhanced oil recovery (MEOR) and bioremediation (Figure 1; Singh et al., 2012; Bachmann et al., 2014). Among the biotechnologies proposed above, those that apply biosurfactants have been the most promising and have received the greatest attention, since biosurfactants' applications can find space in almost all stages of the oil production chain (Silva et al., 2014).

## Biosurfactant characteristics conducive to use in petroleum industry

Surfactants are amphipathic compounds with both hydrophilic and hydrophobic moieties that preferentially partition at the interface between different phases; gas, liquid and solid, and with liquids of different polarities (oil/water and water/oil) and hydrogen bonding. These molecules reduces the surface and interfacial tension, conferring many properties such as detergency, emulsifying, foaming, and dispersing, making them versatile process chemicals (Joshi and Desai, 2010; Silva et al., 2014). Petroleum industry mostly employs petrochemical-based synthetic surfactants as mobilizing agents in their activities (Hazra et al., 2012; Silva et al., 2014). However, demands for sustainable technologies have driven the search for natural, environmental friendly and biodegradable compounds.

Biosurfactants are mainly produced by microbial cultures grown on water immiscible substrates, therefore allowing access to these hydrophobic substrates (such as hydrocarbons) and are generally classified into low molecular-mass molecules (lipopeptides, glycolipids) and high molecular-mass polymers (polymeric and particulate surfactants) (Kapadia and Yagnik, 2013). These molecules offer several advantages over chemical surfactants, such as environmental compatibility, low toxicity, biodegradability, and maintained activity under extreme conditions of temperatures, salinity and pH values (Kapadia and Yagnik, 2013; Santos et al., 2013; Silva et al., 2014). These traits contribute to the relevance of biosurfactants to different industries, especially in the oil industry which has many adverse processes conditions (Silva et al., 2014). Most successful biosurfactants applications that managed to reach the market has been mainly driven by economical production process and cost effectiveness (Banat et al., 2010). This has been facilitated by the lower purity specifications required for such applications, eliminating the purification downstream processing steps which often represent almost 60.0% of the total production costs (Sarubbo et al., 2015). High production cost of biosurfactants has been a major constraining factor that hampers its market growth. Substrate composition accounts for up to 50.0% of the total production costs, the choice of low-cost alternatives therefore is important to the overall economics. Fortunately, biosurfactants can be produced from economical renewable agricultural resources and waste products that can significantly decrease the cost (Helmy et al., 2011; Rufino et al., 2014).

Among the main companies in the global biosurfactants market are Jeneil Biotech, Ecover, Soliance, Saraya, MG Intobio and AGAE Technologies with potential targeted markets covering North America, Europe and Asia-Pacifc (Sajna et al., 2015). The most successful efforts to bring biosurfactant into industrial scale were carried out by Jeneil Biosurfactant Co. (Saukville, Wisconsin) who has successfully developed a production process for rhamnolipids based biosurfactant with a capacity to carry out fermentation processes in batches up to 20,000 gallons (Rufino et al., 2014). Table 1 summarizes commercial manufacturers of different types of biosurfactants and their potential uses in the petroleum industry.

**Table 1:** **Producing companies, types of biosurfactant and potential applications marketed for petroleum industry**.

**Company**	**Biosurfactant**	**Applications**
AGAE Technologies—USA	Rhamnolipids (R95, anHPLC/MS grade rhamnolipid)	Enhanced oil recovery (EOR)
Jeneil Biosurfactant—USA	Rhamnolipids	EOR
Rhamnolipid Companies—USA	Rhamnolipids	EOR
Synthezyme—USA	Sophorolipids	Crude oil emulsification
BioFuture—Ireland	Rhamnolipid	Washing fuel oil tanks
Logos Technologies—USA	Rhamnolipids	EOR
TensioGreen—USA	Rhamnolipids	Petroleum Industry, EOR
Synthezyme—USA	Sophorolipids	Oil and gas
EcoChem Organics Company—Canada	Rhamnolipids-based	Water-insoluble hydrocarbons dispersive agent
EnzymeTechnologies—USA	Bacteria biosurfactant, (unknown)	Oil removal; oil recovery and processing, EOR

Increased environmental awareness has been the main driver for the search for a replacement to chemical surfactants (Marchant and Banat, 2012). According to recent studies, the global market for these “green” alternatives to synthetic surfactants reached US $ 1735.5 million in 2011. In 2013 the total production was approximately 344 kilo tons. Projections for this market share are even more encouraging as it was estimated that by 2018, to reach a value up to US $ 2210.5 million, and in 2020, US $ 2308.8 million when the worldwide market will reach biosurfactants production about 462 kilo tons. The annual average growth rate is expected to reach 4.3% during 2014–2020 (Sekhon et al., 2012; Gudiña et al., 2015; Grand View Research, 2016).

Also according to the same study, Europe was the largest market of biosurfactants consumers with a consumption of 178.9 kilo tons in 2013, representing over 50% of global consumption. North America was the second largest consumer of biosurfactants in the same year, with a participation of more than a quarter. But the Asia-Pacific block had a relatively small market in 2013, but is forecast to gain significant participation over the next 6 years due to the presence of large industries in the region (Grand View Research, 2016).

### Patents on biosurfactants for petroleum industry

The vast structural diversity that characterize biosurfactants leading to a broad range of properties may explain why this group of molecules continues to intrigue scientific interest (Marchant and Banat, 2012; Ławniczak et al., 2013; Luna et al., 2013). This has led to a plethora of patent applications by interested companies and researchers. Several patents have been issued for biosurfactant production from a wide range of microorganisms including *Pseudomonas* spp., *Bacillus* spp., *Acinetobacter* spp. and *Candida* spp. covering many industrial applications (Sachdev and Cameotra, 2013). According to Müller et al. (2012), patents search using the European Patent Office for the terms “biosurfactant”, “rhamnolipid”, “sophorolipid” and “mannosylerythritol lipid” showed a strong increase in number starting from the year 2000. Data showed >250 patents were issued worldwide on biosurfactants and bioemulsifiers with 33% related to the use of petroleum, followed by 15% for cosmetics, 12% for use as antimicrobial agent and biomedical applications and 11% in uses related to bioremediation. Sophorolipids, surfactin, and rhamnolipids related patent represented 24, 13, and 12% of the total number of patents respectively this may however be an underestimate since many patents do not describe or specify the producing microorganism, referring to the general description of a selected biosurfactant (Shete et al., 2006; Reis et al., 2013; Randhawa and Rahman, 2014).

Patents filed in relation to the petroleum industry have been mainly related to uses linked to their properties including wetting, emulsification, phase separation, solubilization, foaming, de-emulsification, corrosion inhibition, and viscosity reduction of heavy crude oils. These patents outline methods and compositions to facilitate the combustion and transportation of highly viscous hydrocarbon-in-water emulsions and in particular, bioemulsifier-stabilized emulsions of hydrocarbon-in-water (Shete et al., 2006). Other patented applications includes using in separating hydrocarbon values from tar sands (Zajic and Gerson, 1981), crude oil recovery from reservoir by MEOR method (Sheehy, 1992), use as bioemulsifier to stabilize hydrocarbons (Hayes et al., 1988), cleaning of oil-contaminated tankers, transportation of heavy crude, recovery of oil from sludge of oil storage tanks (Bachmann et al., 2014) among many other applications. Table 2 lists some of the important patents of bioemulsifiers and biosurfactants in the petroleum industry.

**Table 2:** **Patents issued on the application of biosurfactants relevant to the petroleum industry**.

**Biosurfactants /Organisms**	**Title of Patent**	**Patent No**.	**Author and Year**	**Applications**
Glycolipids	Method and installation for flooding petroleum wells and oil-sands	CA 1119794	Wagner et al., 1982	Recovery of oil from an oil well or oil sands
Biosurfactant-producing microorganisms mixtures	Enhanced oil recovery process using microorganisms	US 4450908	Hitzman, 1984	Enhanced oil recovery
Biosurfactant-producing endogenous microorganisms	Recovery of oil from oil reservoirs	US 5083610	Sheehy, 1992	Oil recovery
Injecting microbial nutrients to stimulate biosurfactant production	Nutrient injection method for subterranean microbial processes	US 5083611	Clark and Jenneman, 1992	Enhanced oil recovery (MEOR).
Lipopeptide	Biosurfactant and enhanced oil recovery	US 4522261	McInerney et al., 1985	Oil recovery
Mixture of microbes, enzymes, surfactants and chemicals.	System and process for in tank treatment of crude oil sludges to recover hydrocarbons and aid in materials separation	US 6033901	Powell, 2000a	Removing of crude oil sludge from oil tank
Treatment fluid containing biosurfactant	System and process for in tank treatment of crude oil sludges to recover hydrocarbons and aid in materials separation	US 6069002	Powell, 2000b	Recover of hydrocarbon
Any biosurfactant producer	Extraction of bitumen from bitumen froth and biotreatment of bitumen froth tailings generated from tar sands	CA 2350907	Duyvesteyn et al., 2000	Extraction and recovery of bitumen
Surface-active agents by exogenous microorganisms	Methods for improved hydrocarbon and water compatibility	US 7992639	Fallon, 2011	MEOR
Stimulation of bacteria with nutrients for production of surfactants	System and method for preparing near-surface heavy oil for extraction using microbial degradation	US 7922893	Busche et al., 2011	MEOR
Consortium including surfactant producer bacteria	Biological enhancement of hydrocarbon extraction	US 7472747	Brigmon and Berry, 2009	MEOR
Viscoelastic surfactants	Bacteria-based and enzyme-based mechanisms and products for viscosity reduction breaking of viscoelastic fluids	US7052901	Crews, 2006	MEOR
Microbial consortia	Process for stimulating microbial activity in a hydrocarbon-bearing, subterranean formation	US 6543535	Converse et al., 2003	MEOR

## Main applications of biosurfactants in the petroleum industry

Biosurfactants have a wide range of biotechnological applications in the petroleum industry. All the operations including exploration and production of oil, refining, transportation, product handling, oil waste management, and responses dealing with accidental pollution or release incidents can be improved, optimized or augmented by the use of some kind of biosurfactant. Table 3, adapted from Silva et al. (2014), presents a list of biosurfactant applications in the four main activities carried out by oil industry.

**Table 3:** **Biosurfactants' applications within the main four petroleum production processes**.

**Step in petroleum production chain**	**Applications**
Extraction	Reservoir wettability modification
	Oil viscosity reduction
	Drilling mud
	Paraffin/asphalt deposition control
	Enhanced oil displacement
Transportation	Oil viscosity reduction
	Oil emulsion stabilization
	Paraffin/asphalt deposition
Oil tank/container cleaning	Oil viscosity reduction
	Oily sludge emulsification
	Hydrocarbon dispersion
Oil waste treatment	Solubilization and mobilization oil

The mechanism behind biosurfactant-enhanced removal and recovery of oil has been proposed to take place through solubilization, mobilization, or emulsification, increasing the area of contact of hydrocarbons (Joseph and Joseph, 2009; Santos et al., 2016). Solubilisation capacity measures a surfactant's ability to increase the solubility of hydrophobic components in an aqueous phase. A significant increase in this capacity occurs when micelles are formed as a result of the partitioning of the hydrocarbon in the hydrophobic part of the micelles. In such a process, higher concentrations of biosurfactants are usually required as hydrocarbon solubility wholly depends on the biosurfactant concentration. Mobilization on the other hand involves both displacement and dispersion. Displacement occurs when hydrocarbon droplets are released from the porous medium as a result of the reduction in interfacial tension. It can also occurs when entrapped hydrocarbon undergoes displacement when sufficient reduction of the interfacial tension between the aqueous and oil phases takes overcoming the capillary forces that cause the formation of residual saturation. Displacements therefore are only related to the interfacial tension between aqueous and hydrophobic phases and not emulsion formation. Dispersion in comparison is a process by which hydrocarbons are dispersed into aqueous phases due to emulsions formation and therefore is linked to both the surfactant concentration and interfacial tension (Sarubbo et al., 2015; Santos et al., 2016).

### Biosurfactants for extraction of crude oil

Oil production strategies traditionally consist of primary depletion followed by secondary recovery and in some cases tertiary recovery processes. In the primary recovery, the initial oil is extracted under natural pressure often only recovering 10–20% of the original oil in place (OOIP; Elraies and Tan, 2012; Bachmann et al., 2014). When oil yields fall due to natural pressure reductions in a reservoir's, secondary recovery technologies are used through either water and/or gas injection. Secondary recovery can lead to an increase of total recovery up to 40–50% of OOIP (Bachmann et al., 2014). Approximately half of the oil in the reservoir remains trapped in small pores of the rock formation. Poor displacement efficiency is attributed to the high forces of capillarity due to surface and interfacial forces, viscosity forces and reservoir heterogeneities (Elraies and Tan, 2012; Santos et al., 2016). Tertiary or enhanced oil recovery methods include chemical and or thermal treatment technologies. Thermal processes are the most common through steam, hot water or combustible gas injection to elevate the temperature of oil and gas in the reservoir facilitating their flow to the production wells. Chemical processes consists of injecting hydrocarbon solvents, surfactants, gas, or combinations thereof to mobilize the residual oil through lowering interfacial tension between oil and water (Elraies and Tan, 2012; Bachmann et al., 2014). This technology is however quite expensive as well as environmentally hazardous which led to the search for eco-friendly and cost-effective alternatives to both thermal and chemical EOR methods (Perfumo et al., 2010).

**Figure 2:**
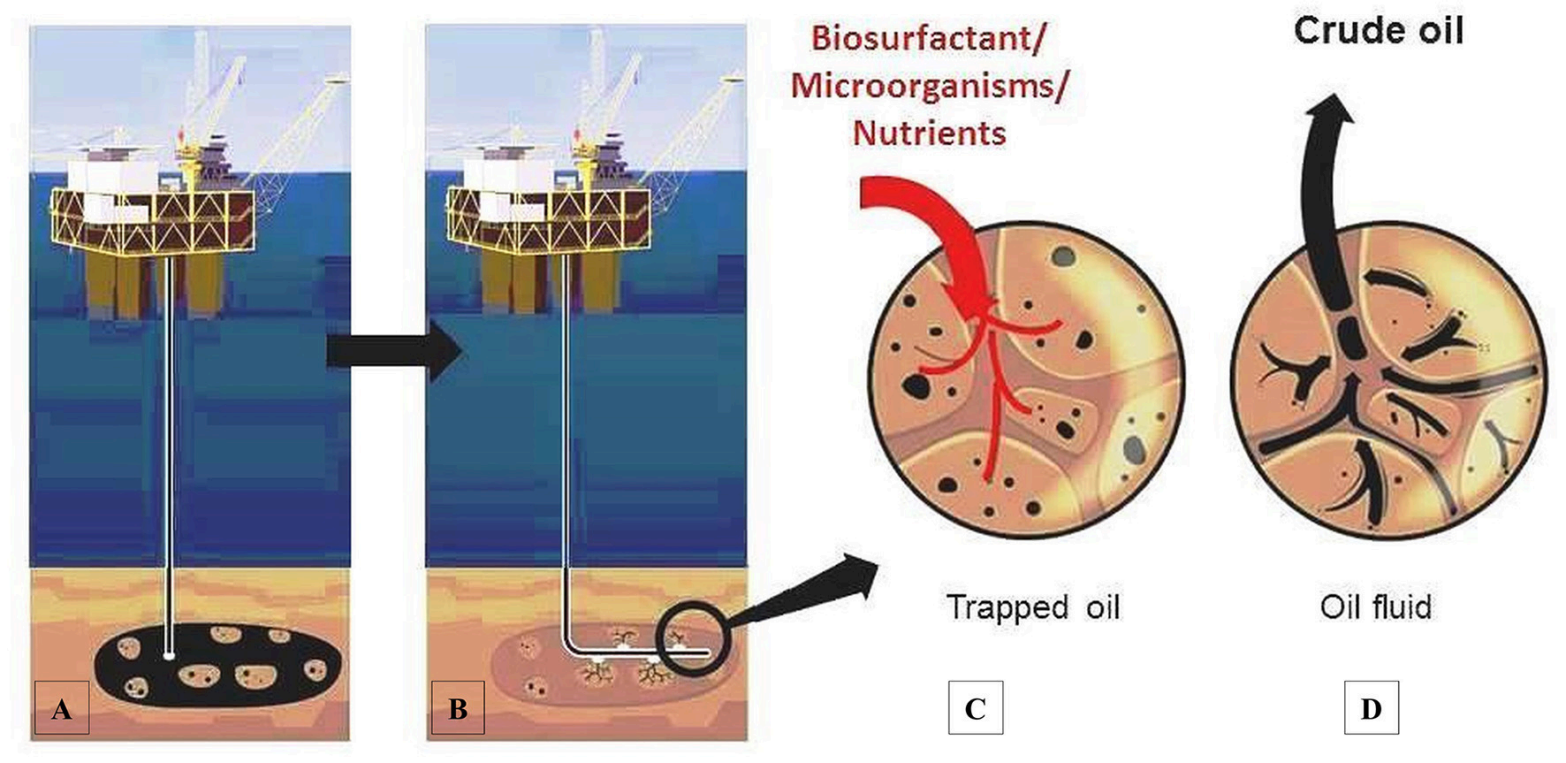
**Process of microbial recovery of crude oil using biosurfactant**. **(A)** Oil extraction using natural pressure of the reservoir. **(B)** Oil well pressure decreased. **(C)** Main strategies of biosurfactant used to the oil release. **(D)** Oil well pressure restored facilitating oil extraction.

MEOR is the tertiary recovery of oil in which microbes or their metabolic products are used to enhance recovered residual oil. It usually is less-expensive when compared to chemically-enhanced oil recovery particularly when microorganisms are used to produce sufficient products such as polymers and biosurfactants starting with low-cost substrates raw materials (Sarafzadeh et al., 2014; Silva et al., 2014). Biosurfactants mainly improve hydrocarbon mobilization thereby enhancing crude oil recovery from reservoirs (Perfumo et al., 2010). There are three main strategies for biosurfactants use in MEOR as shown in Figure 2 they include:

1. Production *ex situ* in industrial setting using bioreactors (batch or continuous culture) followed by subsequent injection into the reservoir along with the water flood (otherwise known as *ex situ* MEOR) (Al-Bahry et al., 2013; Bachmann et al., 2014). Of course biosurfactant production is dependent on the medium composition under controlled setting which is also important for surface-active agent production by the exogenous mixed populations of microorganisms growing *in situ* or added in injection flood waters containing hydrophobic substrate. Excess of carbon/energy source promotes the production of surface-active agents (Fallon, 2011).
2. Microbial augmentation through injecting biosurfactants producing microorganisms at the cell/oil interface within the reservoir formation. This introduces metabolically active cells into the reservoir to allow *in situ* spreading (Al-Bahry et al., 2013; Bachmann et al., 2014). These microbial cells would play a significant role in the surface interactions at interphases between oil and water where they usually prefer to be. It has been reported that at the oil/water interphase, the formed emulsions are proportional to the total biomass produced with increased quality of emulsion at higher quantity of biomass (Bachmann et al., 2014).
3. Nutrients augmentation; injecting essential elements (with or without growth inhibitors for unwanted type of microbial strains) into the reservoir to stimulate the growth of desired indigenous microorganisms producing biosurfactant. Microbial population grows exponentially under favorable conditions producing metabolic products and gases to increase residual oil mobilization within the oil well (Al-Bahry et al., 2013; Bachmann et al., 2014).

All the above strategies increases petroleum yields from a depleted reservoir by decreasing oil-rock surface and interfacial tension and reducing the capillary forces which may impede oil movement through the rock pores. Biosurfactants also enhances the formation of stable water-oil emulsions and the breakdown of the oil film in the rocks which is important for a maximizing oil extraction ultimately extending the reservoir life time (Korenblum et al., 2012; Al-Bahry et al., 2013; Bachmann et al., 2014). The application of MEOR technology however has some disadvantages which includes increased corrosive action against nor resistant equipment due to the introduction of air deployed in aerobic MEOR or logistical problems encountered when high nutrients additives through down-hole piping. Limitations can also be encountered in providing positive pressure to maintain allochthonous microorganisms introduced in the field to produce biosurfactants to enhance oil recovery. Finally most published literature does not include reservoirs physiological and biochemical characteristics of the microflora controlling biological mechanisms nor does it include any details on process economics.

### Biosurfactants uses to enhance crude oil transportation through pipelines

Crude oil is often transported in pipelines from the extraction fields to shipping ports or refineries over long distances. Such transportation particularly for heavy or extra-heavy crudes often represents operational challenges limiting its economic viability. High degree of viscosity due to high paraffins and asphaltenes content in heavy crude oil can decrease its flow which often leads to sludge deposition on the inner walls leading to pressure reductions that ultimately can lead to pipeline plugging problems (Perfumo et al., 2010; Cerón-Camacho et al., 2013). Asphaltenes in particular precipitate in metal pipelines under acidic conditions and in the presence of ferric ions forming asphaltene mud which would deposit in the pipeline obstructing the flow of crude oil. The presence of paraffin in crude oil would decrease the fluidity of oil due to the high content in cyclic hydrocarbons that can solidify and deposit at room temperature, resulting in pipeline blockages in transportation (Assadi and Tabatabaee, 2010). Heating or diluting with solvents, such as xylene and toluene, are traditionally employed to reduce oil viscosity and dissolve any semisolid obstructions, this however of course increases the production cost and generates solvent containing toxic waste residue (Assadi and Tabatabaee, 2010; Mulligan et al., 2014).

A promising technology has been recently developed through the production of a stable oil-in-water emulsion using bioemulsifier biosurfactants to facilitate oil mobility. Such bioemulsifiers are high-molecular weight biosurfactants with different properties compared to low molecular weight glycolipids and lipopeptides. They have a great capacity to stabilize oil-in-water emulsions, but are not effective in reducing interfacial tensions. They also bind tightly to oil droplets and form an effective barrier that prevents drop coalescence due to the high number of reactive groups in their molecules (Perfumo et al., 2010). Emulsan and its analogs, such as alasan and biodispersan, are certainly the most powerful among the bioemulsifiers synthesized by different *Acinetobacer* strains (Mulligan et al., 2014). Bioemulsifier have been extensively studied and have shown potential applications in the formation of heavy oil-water emulsions useful for viscosity reduction during crude transport in pipelines (Assadi and Tabatabaee, 2010; Perfumo et al., 2010; Mulligan et al., 2014). It was reported that such emulsion can under optimal conditions be transported for 26,000 miles. Once reaching destination, the emulsion can either be utilized directly without dewatering or treated with specific enzymes to break the emulsion before use (Mulligan et al., 2014). Amani and Kariminezhad (2016) investigated removing crude oil from a stainless steel tubing using an emulsan type biosurfactant produced by *Acinetobacter calcoaceticus* PTCC1318 and reported successful tube cleaning at the room temperature and suggested suitability for use in pipeline transportation.

The difficulties encountered with such applications however can include the need for high volume or concentration of active materials to be added, or ensuring mixing and continued high pressure into such pipelines. Other concerns may be historical deposition of blockages of transporting pipelines that may need physical clearing methods or use of this technology as a preventative measure to combat such deposition or blockage of new commissioned pipelines.

### Biosurfactants use in oil storage tank cleaning

Large amounts of oil are stored in oil tanks in refineries or transported by oil tankers, barges, and trucks over extended periods. Most such storage tanks and containers are subject to regular cleaning and or maintenance schedule which has often becomes an increasing problem involving hazardous practices and or generating large amounts of hazardous waste (Perfumo et al., 2010; Matsui et al., 2012; Mulligan et al., 2014). The oil sludge fractions that build up at the walls and bottom of the storage tanks are also highly viscous or semisolid and cannot be removed by conventional pumping. The removal of this sludge materials are often carried out manually and may involve the use of steam or hot water or solvents and are hazardous, both time and labor intensive, expensive and usually results in the production of large amounts of waste material for disposal (Perfumo et al., 2010; Matsui et al., 2012).

The use of biosurfactants for cleaning oil storage sludge tanks was proposed for the first time in 1981 as an alternative to traditional methods (Gutnick and Rosenberg, 1981). Ten years later Banat et al. (1991) described microbial biosurfactants applications in oil storage tank cleaning up technology. A field trial was carried out at the Kuwait Oil Company demonstrated that the biosurfactants can effectively drive the cleaning activity of the storage tank. This was carried out through the addition of two tons of rhamnolipid biosurfactants containing culture broth and through energy input to create a liquid vortex within the tank continuously for 5 days at ambient temperatures of 40–50°C. This effectively lifted and mobilized oil sludge from the bottom of the tank and solubilized it within the formed emulsion. The treatment technology recovered 91% of hydrocarbons in the sludge and the value of the recovered crude was estimated to cover the cost of the cleaning operation (Galabova et al., 2014; Mulligan et al., 2014). The recovered hydrocarbon had excellent properties and could be sold after being blended with fresh crude (Banat et al., 1991). An improved process encompassing this technology was patented in 2004 by Idrabel Italia (Italy) and Jeneil Biosurfactant Company (United States). As a result of the implementation of the proposed process, the recovery of oil has generally been > 90% of the total sludge volume with a reduction of material to be disposed of to values <5% of the original sludge volume (Galabova et al., 2014). It is however important to note that the application of these technologies requires significant engineering expertise to ensure the delivery of the active ingredient and energy input that is required for mixing tanks content all of which within a highly controlled and regulated environment of in terms of safety provision and consequences or accidental hazardous practices in oil refineries and installations.

Diab and El Din (2013) also evaluated the effect of *P. aeruginosa* SH 29 biosurfactant in cleaning oil-contaminated vessels. They reported successful oil removal from the vessels bottom and walls within 15 min of application under laboratory conditions, floating as a supernatant distinct phase. They concluded suitability of the product and process for use in vessels used for the transportation and storage of crude oil. Similar observations were reported by Rocha e Silva et al. (2013) using biosurfactant from *Pseudomonas cepacia* CCT6659 for cleaning oil covered beaker walls. Matsui et al. (2012) also carried out a successful oil tank bottom sludge cleaning process using a biosurfactant produced by an actinomycete *Gordonia* sp. and reporting dispersion activity greater than that achievable with a chemical or plant-derived surfactant. Most industrial operators currently working in the field of dispersion and oil sills control have highly effective chemical dispersants for deployment when needed, all of which have official approval. For biosurfactants to replace these chemical dispersants they have to present significant clear advantages in addition to biodegradability, and at present these are probably limited, since biosurfactants are less efficient dispersants than current chemical products and are certainly more expensive to produce on a large-scale.

### Biosurfactants for oil waste treatment

During oil exploration, storage, transport and refining processes a considerable amount of oily sludge is generated by the petroleum industry (Hu et al., 2013). The disposal of such residues has always been a major issue faced by petroleum industries (Joseph and Joseph, 2009). For example, the annual output of oil sludge in China's refineries was estimated to approximately be one million tons, mainly derived from the cleaning process of oil storage tanks (Liu et al., 2011). In India, about 28,000 tons of oily sludge are generated by the refineries industries per annum (Joseph and Joseph, 2009). Oily sludge is a complex emulsion of various petroleum hydrocarbons containing solid particles, water and heavy metals that effective treatment methods have become a highly sought after technology attracting widespread attention (Hu et al., 2013).

Different technological options have been adopted by petroleum refineries worldwide to manage generated wastes during crude refining and stocking (Joseph and Joseph, 2009). Typically, various physical and chemical processes such as solvent extraction, dewatering, and incineration, stabilization, pyrolysis, washing with hot water or surfactant, and biodegradation are among the most common oil sludge handling techniques. Such methods are often expensive and requires complex equipment increasing cost and complexity (Guolin et al., 2011). Biological methods may be considered more suitable due to their less hazardous and more selectivity to specific reactions (Assadi and Tabatabaee, 2010). Various investigations in laboratory, pilot and field scale have been carried out to use biosurfactants in oily sludge treatment and have reported obtaining higher oil recoveries using biosurfactants (Pornsunthorntawee et al., 2008; Hu et al., 2013).

Lima et al. (2011) evaluated the removal of oily sludge through the use of biosurfactants obtained from five bacterial isolates from oil contaminated sites. Biosurfactants use led to a reduction in viscosity and promoted the formation of oil-water emulsions leading to easier sludge pumping and emulsion breaking for better crude oil recovery. The process was highly efficient for oil recovery resulting in up to 95.0% reduction in sludge volume. In laboratory and pilot-scale experiments, Yan et al. (2012) investigated the use of a rhamnolipid produced by *Pseudomonas aeruginosa* F-2 to recover oil from refinery oily sludge reporting up to 91.5% oil recovery during field pilot-scale studies.

Petroleum industry unavoidably generates large volumes of oily wastewater which has become an urgent challenge for most oilfield and petroleum company focusing attention toward efficient treatment techniques (Yu et al., 2013). Separation technologies such as centrifugation, ultrafiltration, decantation, flotation, and flocculation are examples of physical/chemical processes effectively used for the separation of oil-water mixtures (Painmanakula et al., 2010). Coagulants of chemical origin are usually used to improve the efficiency of separation of oil-water (Liu et al., 2010). Biosurfactants however are promising coagulants and/or dispersants capable of increasing the efficiency of these techniques. For instance, Rocha e Silva et al. (2015) investigated the removal of the emulsified oil products from water in a pilot scale by dissolved air flotation and reported increasing separation efficiency from 80.0 to 98.0% in the presence of biosurfactants.

It is important to note that although there are many reports on successful application applications of biosurfactants in such bioremediation processes, several cases of little or no effects of biosurfactant use in these activities have been reported (Franzetti et al., 2011). This may be mainly due the complex interactions occurring within this environment between the cell surfaces, the amphiphilic and the abiotic environment. A more detailed understanding of the natural roles and effects of biosurfactants on biological and abiotic compartments is therefore necessary to consider them as a fully reliable agents for enhancing bioremediation.

### Biosurfactants as demulsifying agents

Oilfield emulsions represent one of the major problems for the petroleum industry and are generated at various stages of petroleum exploration, production and recovery. Such emulsions are often complex and are a result of the prevalence of amphiphilic molecules within the oil such as the resin fraction containing naphthenic acids and asphaltenes in addition to fine solids such as clays, scales, and wax crystals (Assadi and Tabatabaee, 2010; Reis et al., 2013). The water present in oil emulsions may originate from water or steam injected to improve oil recovery or water added during de-salting operations and need to be separated out by breaking the emulsion prior to refining. The presence of water can cause problems including corrosion, scale formation, sludge accumulation in storage tanks, reduced distillation efficiency, and altered viscosity and flow properties (Mohebali et al., 2012). Breaking the emulsion (de-emulsification) takes place through the disruption of the thermodynamic conditions at the interface leading to the disruption of the stable surfaces between the bulk and the internal phases. It is, therefore, an important process before downstream oil processing, as emulsifying agents can hinder the production processes (Satpute et al., 2010). De-emulsification is a challenging process that is usually carried out by physical treatment methods including centrifugation, heat treatment, electrical treatment and/or through chemicals and as such are capital intensive and constitute a disposal problem as most chemical de-emulsifier(s) have the potential to cause environmental problems (Assadi and Tabatabaee, 2010; Mohebali et al., 2012; Reis et al., 2013).

Microbial de-emulsifiers generally have low toxicity and are biodegradable and often have unique characteristics that cannot be matched by chemically synthesized alternatives (Mohebali et al., 2012). Biological de-emulsifiers also can replace the use of chemical de-emulsifiers *in situ* which reduces the need to transport oil emulsion for treatment and provides a more environmentally-friendly solution. They are also easier to remove and recover at the end of the process (Reis et al., 2013). Microorganisms exploit the hydrophilic/hydrophobic nature of biological surface active compounds to disrupt the emulsions. Glycoproteins, glycolipids, phospholipids and polysaccharides are such microbial metabolites capable of displacing emulsifiers from the oil-water interface. Some researchers have also reported that microbial de-emulsification abilities are phenomena associated with microbial whole cells including those of *Acinetobacter* sp., *Pseudomonas* sp., *Nocardia* sp., *Bacillus* sp., *Rhodococcus* sp. *Corynebacterium* sp., and *Micrococcus* sp. (Assadi and Tabatabaee, 2010; Mohebali et al., 2012; Reis et al., 2013).

Chirwa et al. (2013) compared the de-emulsification and separation ability of oil and sludge using either commercial sodium dodecyl sulfate (SDS) surfactant with that from a biosurfactant and reported a slower recovery with biosurfactant compared to SDS yet strong feasibility for using biosurfactants for removal and recovery of oil from waste sludge. Most of the literature testing demulsifying capabilities and many other biosurfactant related activities have used crude biosurfactant extractions which not only have some other components within, but contains mixtures of biosurfactant congeners that often has different characteristics and properties. One feature of microbially produced biosurfactants is that they are synthesized as a mixture of different congeners with varying bioactivity. For many applications this is a big disadvantage and considerable downstream processing would be required to produce a product that could be used in the formulation of a consumer product. The ability to purify the products and separate such congeners we expect would significantly improve our knowledge and outcomes in this regards.

### Biosurfactants as anti-corrosive agents

Corrosion represents a major problem for the petroleum industry. All equipment used in oil wells refineries, petrochemical plants and transport are susceptible to corrosion with consequent negative effects on investment within the petroleum sector (Kanicky et al., 2002; Abbasov et al., 2015; Noor El-Din et al., 2016). Corrosion often starts with the adsorption of protons on metallic surfaces and an irreversible electrochemical reaction with the metal atoms. The metallic cations either dissolve in the aqueous phase or react with anions such as sulfur therefore exposing more metallic surface for subsequent attacks (Kanicky et al., 2002). Such corrosion problems have been long known to be associated with naphthenic acid and sulfur compounds constituents of crude oil refining products (Saji, 2010).

Corrosion inhibitors have been the focus of research for many years as the most practical methods for prevention. Controlling corrosion in oil field is quite complicated and requires specialty inhibitors depending on the area of application such as wells, refineries, pipelines, recovery units, pipelines storage tanks, etc. Such inhibitors can be inorganic or organic chemicals surfactant or mixed components inhibitors (Saji, 2010; Malik et al., 2011). Synthetic surfactants are usually used to control corrosion due to their ability to affect the properties of surfaces and interface mostly through adsorption to the metal surface reducing the chance of corrosion initiation. Most such chemicals however, have risks and hazardous effects to people and the environment. An alternative is the use of biosurfactants to replace the chemically synthesized surfactant compounds (Malik et al., 2011; Korenblum et al., 2012).

Most of the biosurfactants exhibit anti-corrosion properties and have a great potential for such use through conditioning metals surfaces to delay the corrosion process (Korenblum et al., 2012; Araujo and Freire, 2013). Metal corrosion leads to the formation of corrosion products and release of energy. The most protected surfaces against corrosion are those with lower free energy. When surfaces interact with H^+^ ions they tend to become more hydrophilic which may initiate the corrosion process. When surfaces however are conditioned with biosurfactants a film of these molecules attach to the surface, orienting the hydrophobic tail to the external environment while hydrophilic head to the surface, maintaining the surface protected from interaction with O_2_ and H^+^ ions, reducing corrosion (Malik et al., 2011; Araujo and Freire, 2013). In a study of corrosion behavior of metal surface carried out by Dagbert et al. (2006), he reported that the presence of biosurfactant produced by *Pseudomonas fluorescens* significantly delayed the corrosion of the AISI 304 stainless steel surface.

## Other applications for biosurfactants in the oil industry

### Biosurfactant for control of sulfate reducing bacteria (SRB)

SRB are a group of anaerobic bacteria that use sulfate (${\text{SO}}_{4}^{-}$) as a final electron acceptor instead of oxygen during anaerobic respiration and are known to cause oil reservoir souring and microbial induced corrosion making them to be considered undesirable and harmful for the oilfield production process (Dinh et al., 2004; Hubert et al., 2005; Song et al., 2014). Oilfield souring occurs as a result of H_2_S and sulfides ions production, which occurs when the reservoirs are subjected to water flooding during secondary oil recovery. H_2_S can also accelerated corrosion rates (Gouda et al., 1993). SRBs' biomass and sulfide metals ions can also decrease the efficiency of secondary oil recovery due to reservoir plugging (Nemati et al., 2001), in addition to the toxic and explosive nature of hydrogen sulfide when mixed with air (Gaathaug et al., 2014).

Although SRB are mainly known to use different low molecular organic compounds such as simple organic acids or alcohols and often H_2_ for growth while reducing ${\text{SO}}_{4}^{-}$ to H_2_S, recent studies have shown that hydrocarbons in petroleum may also serve as electron donors for SRBs (Nemati et al., 2001; Song et al., 2014). When seawater or other waters containing sulfate are introduced into oil reservoirs, SRBs intensify the souring process though sulfate reduction, to sulfide while oxidizing organic electron donors present in the crude oil (Korenblum et al., 2012). Naturally souring decreases the value of the produced oil and increases the corrosion risk, increasing, thus, the total cost of oil production (Nemati et al., 2001; Hubert et al., 2005). Microbial corrosion represents some 10% of all damages to metals and non-metals (Dinh et al., 2004). Severe microbial corrosion on petroleum reservoirs occurs under anaerobic conditions and *Desulfovibrio* species are conventionally regarded as the main culprits of corrosion to oil transport equipment, including pipelines (Korenblum et al., 2012; Song et al., 2014). This process often occurs within microbial biofilms which starts with the adhesion in which hydrophobic interactions between the abiotic surface and the microorganism and progress to maturation in time leading to metal pitting (Sherry et al., 2013).

Different approaches can be used to control SRBs proliferation mainly through the use of biocide among which glutaraldehydes cocodiamines and molybdates (Nemati et al., 2001). However, both the cost and the environmental impact of using these compounds are usually high (Korenblum et al., 2012) as they can lead to the emergence of biocide-resistant SRBs and do not effectively penetrate biofilms within reservoirs or on metal surfaces in addition to causing corrosion themselves at high concentrations (Hubert et al., 2005).

Therefore, the provision of alternative sources to chemical biocides is desired by the oil industry. Recently, biosurfactants have been shown to be potential alternatives to chemical biocides and as surface coating agents to prevent SRBs growth. Their antimicrobial activity and surfactant properties increase the osmotic pressure within the cell causing leakage of the intracellular contents (Korenblum et al., 2012). El-Sheshtawy et al. (2015) assessed the inhibitory potential of biosurfactant from *Bacillus licheniformis* to SRBs growth and reported some antimicrobial activity against the growth of different strains of SRB and a complete inhibition of SRB growth after 3 h exposure to 1.0% crude biosurfactant.

### Biosurfactant for extraction of bitumen from tar sands

Tar sands are sedimentary rocks that contain bitumen and other heavy petroleum fractions and are usually the product of biodegradation and chemical changes due to bacteria degradation and water washing (Spirov et al., 2013). The largest tar sands deposits are in Canada, USA, Venezuela, Madagascar and Russia and the biggest producer of synthetic oil from tar sands is Canada. In 2010, 55% of its tar sands production was from mining operations with a maximum burial depth of 75 m while *in situ* operations produced, the other 45% had deeper depths. The proportion of non-upgraded bitumen exports is projected to increase from 42% of total production in 2009, to 52% by 2019 (Spirov et al., 2013; Rudyk and Spirov, 2014).

The recovery of bitumen from tar sand is a difficult process due to its high viscosity which is typically reduced by steam (300–340°C), solvents or caustic soda injections into the sands. These processes require more water and need larger amounts of energy than conventional extraction methods (Spirov et al., 2013). Biosurfactants have been tested for bitumen extraction from tar sands and have shown effectiveness in reducing the interfacial tension between oil and water *in situ* while acting on solid-liquid interfaces. These proprieties can be used for viscosity reduction of the oil, removing water from emulsions prior to processing and releasing bitumen from tar sands. Such process can be carried out at lower temperatures and without requiring the use of caustic soda both of which are considered advantageous (Duyvesteyn et al., 2000; Oliveira et al., 2015). Moreover, bitumen froth can be extracted from tar sands using a water process which involves the biotreatment reducing waste by-products (Mulligan and Gibbs, 1993; Shete et al., 2006). The type of microorganisms used for this purpose included *Bacillus megaterium, Arthrobacter terregens, A. xerosis, Corynebacterium lepus, C. xerosis, Pseudomonas asphaltenicus, Nocardia petrophilia* and *Vibrio ficheri* (Shete et al., 2006).

Cooper and Paddock (1984) tested glycolipids produced by the yeast *Torulopsis bombicola* ATCC 22214 in the release of bitumen from tar sand and reported effects on liquid-liquid and solid-liquid interfaces which caused significant release of bitumen from the sand. Zajic and Gerson (1978) evaluated the performance of microbial surfactants for the recovery of bitumen from Athabasca tar sand, in northeastern Alberta, Canada. These surfactants were produced by hydrocarbon fermentations of five different strains (*Corynebacterium* sp. OSGBl, *Pseudomonas* sp. Aspha 1, *Candida lipolytica* GA, *Vibrio* sp. Chry-B and *Corynebacterium* sp. CD1). These microbial surfactants compared well with synthetic surfactants and proved to be effectives in tar sand separation by a cold-water extraction process to cause flotation of the bitumen or to cause removal of sand and clay from the bitumen.

## Future prospects for biosurfactants in the petroleum industry

### Biosurfactant for fuels formulation

One of the unexplored area for potential biosurfactant applications in the petroleum industry is possible use in the formulation of emulsified fuels (Youssef et al., 2009; Perfumo et al., 2010). Emulsified fuels are mixtures that includes surfactants that facilitates the formation of a stable emulsion of the water or other substances within the fuel phase and a variety of additives such as detergents, anti-foaming agents, lubricity enhancers, anti-rust agents, ignition improvers and metal deactivators (Coleman and Sibley, 2003; Dantas Neto et al., 2011).

Diesel fuel blended with water is a well-known emulsified fuel currently applied worldwide for public transport fleets, locomotives, marine engines and heat generators in industrial settings. In addition to cost saving such fuels improve combustion efficiency, do not need engine modification and effectively reduces carbon monoxide (CO), NO_*x*_, unburned hydrocarbon, particulate matter emission and reduce exhaust gas temperatures and general pollutant emissions (Perfumo et al., 2010; Dantas Neto et al., 2011). Surfactants can stabilize the emulsion ensuring that the finely dispersed water droplets remain in suspension within the fuels preventing phase separation upon long-term storage.

Currently the most used surfactants includes non-ionic and polymeric surfactants such as alcohol ethoxylates, sugar esters of fatty acids, and fatty acids ethoxylates. However, investigations into the possibility of replacing traditional chemical compounds with microbial surfactants to formulate fuel or diesel emulsions have been carried out (Coleman and Sibley, 2003; Perfumo et al., 2010). Leng et al. (2015) successfully tested a biosurfactant rhamnolipids to obtain nano-scaled glycerol/water-in-diesel microemulsions, which can be formed spontaneously with low energy consumption. In addition, the physicochemical properties of glycerol/water-in-diesel microemulsion were similar to those of diesel.

### Recombinant DNA technology to enhance biosurfactant production

Genetic engineering consists in modifying the genetic material of microorganisms of industrial importance to acquire new or enhanced capabilities through recombinant DNA technology. The construction of hyper producing microorganisms to increase the biosurfactant secretion to promote activity and decrease cost is a general aim (Assadi and Tabatabaee, 2010). However, industrial-scale usage of biosurfactants for MEOR still appears to be limited due to high production costs (Banat et al., 2010; Makkar et al., 2011). To reduce this cost it is important to develop mutant or recombinant strains with enhanced production yields (Bachmann et al., 2014), or with an ability to selectively produce particular effective congeners of biosurfactants which are often a mixture of closely related products. Biosurfactants producers could also be engineered to be resistant to process conditions generally found in the petroleum industry. An alternative is to isolate new gene sequences from extreme environments similar to ones that might be encountered in oil reservoirs such as high salt concentration, high temperatures, and extreme pH values. For example, alkaliphilic halophiles microorganisms can be found in hypersaline soda lakes such as Lake Magadi in Kenya, Wadi Natrum lakes in Egypt and Soda lakes in China; genes from such isolates may then be transferred into selected biosurfactant producers which can be active and effective under such extreme conditions. Other possibilities include the use genes that code for the production of biosurfactant that are particularly well evolved at elevated temperatures through isolation from high temperature oil reservoirs (Kohr, 2012; Kohr et al., 2016).

## Concluding remarks

It is concluded that advances in oil biotechnology are becoming increasingly evident in recent years and due to the versatility and efficiency demonstrated by many types of biosurfactants in the service of or in processes related to the petroleum industry, they are increasingly gaining recognition and appreciation. These compounds are not only providing supporting roles but are beginning to provide essential roles, making them necessary compounds in petroleum biotechnology. The one major advantage of biosurfactants would be their biodegradability which significantly reduces the environmental impact of these compounds compared to chemical surfactants. It is their other successful applications, however that are becoming recognized and we believe will lead to an expansion in their use within the petroleum industries.

## Author contributions

All authors contributed in this work. DD, RS, VS, LS, IB, RR, and JL designed the project and wrote the manuscript. LS and IB carried out manuscript editing and final improvement.

## Funding

Funding for this study was provided by the State of Pernambuco Foundation for the Assistance to Science and Technology (FACEPE), the Research and Development Program of the Brazilian National Electrical Energy Agency (ANEEL), the National Council for Scientific and Technological Development (CNPq) and the Federal Agency for the Support and Evaluation of Graduate Education (CAPES).

### Conflict of interest statement

The authors declare that the research was conducted in the absence of any commercial or financial relationships that could be construed as a potential conflict of interest.
